# Exploring the impact of colostrum supplementation on athletes: a comprehensive analysis of clinical trials and diverse properties

**DOI:** 10.3389/fimmu.2024.1395437

**Published:** 2024-05-10

**Authors:** Yalçın Mert Yalçıntaş, Barış Baydemir, Hatice Duman, Furkan Eker, Ayşe Bayraktar Biçen, Melih Ertürk, Sercan Karav

**Affiliations:** ^1^Department of Molecular Biology and Genetics, Canakkale Onsekiz Mart University, Canakkale, Türkiye; ^2^Department of Coaching Education, Canakkale Onsekiz Mart University, Canakkale, Türkiye; ^3^Uluova Dairy, Çanakkale, Türkiye

**Keywords:** colostrum, athletes, sport nutrition, disorders, supplementation, health and immunity

## Abstract

Colostrum, an invaluable food produced by mammals during the postnatal period, contains important bioactive components. It is a valuable therapeutic substance that can be used to treat a variety of disorders, in addition to its primary function of providing passive immunity to newborns. Undoubtedly, a strong dedication to intense effort and demanding training schedules is necessary to succeed in today’s sports environment. Peak physical fitness, strategic skill development, and mental toughness are highly valued in the environments in which athletes compete. However, the inherent difficulties brought about by athletes’ intense schedules are matched with the demanding character of modern sports. The intensity of athletic activity frequently provides little time for sufficient relaxation, nutritional preparation, and overall recovery, which can contribute to mental and physical tiredness. Athletes need to develop all-encompassing strategies to overcome these obstacles. These strategies should prioritize self-care and recovery in addition to maximizing training efficiency. The bioactive components of colostrum bring forth various therapeutic effects against the challenges experienced by athletes; including diarrhea, upper respiratory tract infections, muscle injuries, intestinal disorders, etc. This review examined the different therapeutic effects of the bioactive components of colostrum on athletes, the effect of the use of colostrum as a whole on the performance of athletes, and the clinical research conducted in this field. While the majority of studies report positive effects of colostrum, further research is needed.

## 1 Introduction

Colostrum is a distinctive fluid secreted by mammals in the postnatal period that provides a vital source of nutrients for nursing young. This special fluid, which stands out with its rich nutrient composition, has the potential to improve performance, gaining a remarkable place in athlete-oriented research (1, 2). Research in the field of sports underlines the capacity of colostrum to have positive results for athletes (3, 4).

Colostrum contains many biologically active substances. Key components include immunoglobulins, growth factors, lactoferrin(Lf), vitamins, and minerals. These components have the potential to influence athletes’ training performance and recovery processes (5). Colostrum contains growth factors, and in athletes, these properties can support muscle development and provide faster recovery. Additionally, thanks to immunoglobulins, it can strengthen the immune system and protect against diseases that may occur depending on the frequency of training (4, 6).

In recent years, the increased emphasis on sports compared to the past has not only enhanced the pleasure of watching but has also introduced significant challenges. Achieving high effort levels in sports necessitates intensive training, which, over time, becomes a stimulant for performance. However, such rigorous training is also highly susceptible to causing injuries in athletes. Immune suppression and excessive muscle strain contribute to an increased risk of injuries.

At this juncture, Bovine Colostrum (BC) emerges as a crucial consideration for consumption, playing a vital role in preventing injuries and strengthening muscles. BC positively influences muscle and bone development due to the significant amount of growth factors it possesses. Moreover, in cases of potential muscle injuries or severe disability, BC can expedite the treatment process, thanks to its growth factors and other components. It accelerates tissue repair in damaged areas by increasing cell proliferation, preventing athletes from being incapacitated during training and competitions (7). BC consumption offers substantial support against such situations, recognizing that injuries not only harm athletes but also have enduring negative effects on their long-term fitness charts.

**Figure 1:**
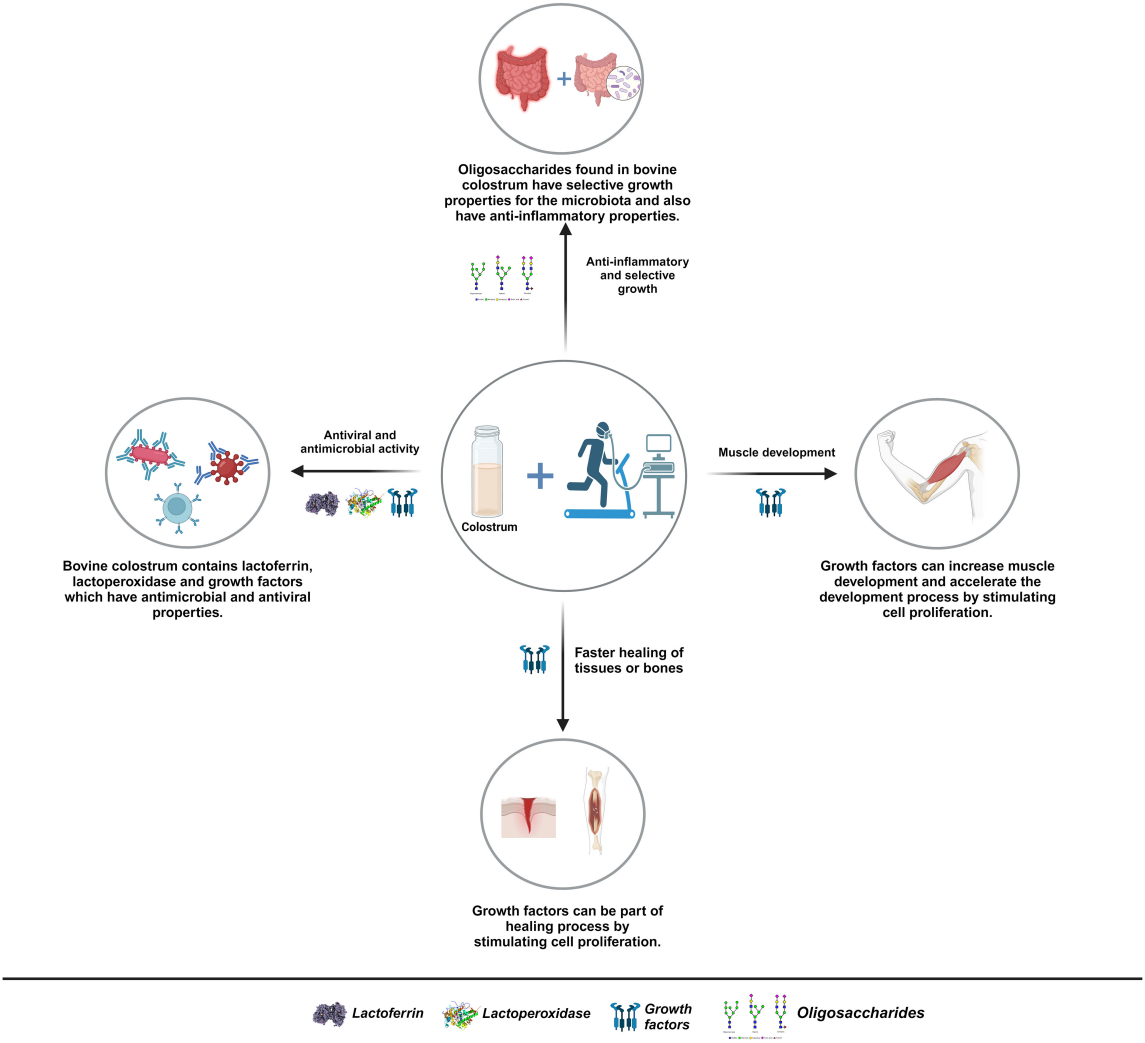
Effects of colostrum on athletes. The consumption of BC can support immune regulatory effects, accelerate the healing process of injuries, selectively promote microbiota growth, enhance bone health alongside muscle development, and prevent inflammation in the intestines. These features, corroborated by clinical research on athletes, could be further explored through additional studies and might yield more pronounced positive effects in the long term if BC (7, 9–11).

According to research, heavy training may result in immunosuppression. Athletes who have reached high fitness levels need to work constantly to maintain their stability, but diseases caused by immunosuppression may affect this stability and cause poor form (8). The bioactive components contained in BC, which will be mentioned in this review, have antimicrobial and antiviral properties with the potential to not only shorten the treatment process but also prevent exposure to diseases after training by providing passive immunity. In addition, BC regulates intestinal permeability with the bioactive components it contains, selectively has positive effects on the microbiota, and provides significant protection against intestinal disorders, which is shown in **Figure 1**. Current research on the use of BC in athletes suggests that this natural fluid may favorably affect sports performance and recovery (12). However, it needs to be emphasized that more extensive research is required. Specific dosage and usage recommendations for the consumption of BC in athletes need to be standardized. It is thought that this review will guide the reporting of positive or negative results in the performance of athletes with the use of BC and current research.

## 2 Bioactive components of colostrum and their effects on athletes

In today’s sports world, high intensity is notably prominent, subjecting athletes to rigorous training to achieve peak performance. Despite the success that heavy and intense training brings, a significant threat looms: the ‘Open Window.’ This condition refers to immune suppression following prolonged and intense exercise (13), leading to upper respiratory tract infections (URTI) that can pose serious challenges for athletes. While URTI may not appear severe, its potential to impact athletes’ performance and result in setbacks during competitions should not be underestimated (9). BC consumption manifests various positive outcomes among different target groups, particularly demonstrating protective effects on athletes. For instance, BC serves as a crucial supplement against diseases due to its immune-supporting components, ensuring stability by preventing performance fluctuations linked to illnesses. The potential of BC to directly influence immunity, attributed to the presence of immunoglobulins, was highlighted in a study showing that even modest levels of BC consumption significantly affected peripheral blood neutrophil phagocytic activity (14). Building on these findings, a study on athletes revealed that post-training BC consumption increased immunoglobulin levels in the body (1). It is evident that BC consumption is associated with a reduced risk of URTI and other ailments in athletes, underscoring its critical role in disease prevention.

On the other hand, BC contains a significant amount of growth factors. More importantly, BC is the only natural source that contains two main growth factors [Transforming growth factors (TGF) and Insulin-like growth factors (IGF)] (15). Growth factors have different functions in the body, but they are of great importance from the athlete’s perspective. They are playing a key role, especially in muscle injuries and open wounds, which are some of the worst situations that can happen to athletes. They support cell proliferation, signaling, and tissue repair (7) (**Table 1**). Thanks to these features, they can enhance the healing process of the damaged area in cases of injury during training or sports. Growth factors can also play an immune-supporting role against pathogens entering the body and have a positive effect on increasing the number of immunoglobulins (27). Barrientos et al. (2008) has supported the idea that growth factors can significantly contribute to the body’s bone and muscle development during the training process (7). They enhance the active development process of the muscles in the area where muscle damage occurs. It has also been stated that some growth factors are good for intestinal health and have positive effects on the microbiota (28, 29). Studies have shown that oral consumption of TGF-β found in colostrum increases Immunoglobulin A (IgA) production in newborns and reduces gut inflammation in premature piglets. These findings are consistent with those from cell culture studies and suggest that orally administered growth factors may have similar effects in living organisms, particularly on gut health and the immune system (30–32). However, further research is needed to determine whether the effectiveness of their positive effects on the body varies depending on the route of consumption.

**Table 1:** Bioactive components in colostrum and their potential effects on athletes.

Component	Concentration (g/L)	Effect	Reference
Protein	250	Increase development and repair in the body.	(16)
Casein	26	Decreasing blood pressure, minimizing the occurrence of blood clot formation and aiding digestion in the stomach.	(16–19)
Immunoglobulin	20-150	Prevents post-exercise illnesses by supporting the athlete’s immunity against post-exercise immune suppression.	(16)
Lactoferrin	1.5	Antimicrobial, antiviral and antifungal properties	(16, 20–22)
Growth Factors	50 μg to –40 mg/L	Cell proliferation, faster healing of wounds and muscle development.	(7, 16)
Lactoperoxidase	0.02	Anti-pathogenic effect and contributes to immunity.	(23)
Oligosaccharides	0.7-1.2	Selective growth of microbiome.	(16, 24–26)

**Table 2:** Clinical applications of colostrum on athletes.

Target group	Dose & duration	Study Design	Effect	Reference
**Athletes**	20 g BC/day Total: 8 weeks	Randomized controlled and double-blind, placebo-controlled study design. Placebo: Whey protein (20g/day in powder form)	Achieved a lean mass gain of +1.49 kg, indicating the potential for increased lean mass in active individuals.	(10)
**Sprinters and jumpers**	125ml BC/day, 8 days of strength and speed training, 13 days apart.	Randomized, double-blind, crossover study design. Placebo: Whey protein (125 ml/day in liquid form)	Increased IGF-I levels in athletes (≈ +5 nmol/l in BC group, ≈ -4 nmol/l in placebo), promoting muscle growth and anabolic effects.	(33)
**35 elite field hockey players**	60 g BC/day Total: 8 weeks	Double-blind, randomized, placebo-controlled study design. Placebo: Whey protein (60g/day in powder form)	Enhanced elite field hockey players’ sprint and jump performance. Colostrum supplementation may enhance sprint performance by 2.3% more than whey supplementation.	(34)
**51 athletes**	60 g BC/day Total: 8 weeks	Randomized, double-blind, placebo-controlled parallel study design. Placebo: Whey protein (60g/day in powder form)	Vertical jump (7370 to 7237 W) and cycle power (1400 to 1311 W) was higher in the BC by 1.5% and 2.3% respectively	(35)
**42 cyclists**	20g BC + 40g whey protein, or 60g BC or WPC (placebo). 8 weeks.	Randomized, double-blind, placebo-controlled study design. Placebo: Whey protein concentration (60g/day in powder form)	The cyclists improved their performance ride time by 134 (20g) and 158 (60g) seconds, when compared to the placebo group which is 37 seconds.	(36)
**29 highly trained road cyclists**	60 g/day Total: 8 week	Double-blind and placebo-controlled study design. Placebo: Whey protein (10g/day in powder form)	Enhanced recovery, potentially increasing performance by up to 5.2% after 8 weeks.	(37)
**13 elite rowers**	60 g BC/day Total: 9 weeks	Randomized, double-blind, placebo-controlled study design. Placebo: Whey protein (10g/day in powder form)	Improved blood buffer capacity (BC group ≈ 40, compared to placebo ≈ 33, in slykes unit), but did not enhance performance significantly.	(38)
**16 athletes**	500 mg BC/day Total: 20 days	Double-blind and placebo-controlled study design. Placebo: Whey protein (500mg/day in powder form)	%75 of the BC group (6) showed reduced intestinal permeability. Meanwhile 62.5% of the placebo group (5) experienced infections.	(39)
**25 soccer players**	3.2 g BC/day Total: 6 weeks	Double-blind, randomized, placebo-controlled study design. Placebo: Whey protein (3.2g/day in capsule form)	Enhanced recovery after intense exercise, affecting biochemical parameters.	(40)
**12 individuals**	20 g BC/day Total: 14 days	Double-blind, placebo-controlled, crossover study design. Placebo: Isoenergetic and isomacronutrient milk protein concentrate (20g/day in powder form)	Placebo group showed higher increase in cell damage marker (I-FABP) than BC group, 194% to 134%.	(41)
**12 athletes**	20 g BC/day Total: 14 days	Double-blind, placebo-controlled, crossover study design. Placebo: Isoenergetic and isomacronutrient milk protein concentrate (20g/day in powder form)	Placebo group showed higher Intestinal permeability increase (2.5-fold) when compared to BC group 80%) Reduced exercise-induced intestinal permeability and enhanced performance while preventing heat stroke.	(42)
**20 athletes**	20 g BC/day Total: 4 weeks	Randomized, double-blind, parallel study design. Placebo: Isoenergetic and isomacronutrient milk protein concentrate (20g/day in powder form)	BC supplementation demonstrated significant effects on exercise-induced changes in innate immunity compared to the placebo group. Four-week BC supplementation countered exercise-induced immunosuppression, enhancing host defense.	(43)
**35 distance runners**	10 g BC/day Total: 12 weeks	Randomized, double-blind, placebo-controlled study design. Placebo: Maltodextrin blend with skim milk (250ml/day in liquid form)	Increased s-IgA levels (79%) , potentially reducing infections in athletes.	(44)
**8 healthy individuals**	60 microgram/kg Total: 2-4h (Has repeated 6 months later)	Crossover, randomized, double-blind study design.	Improved heart function, increased stroke volume (14%), and cardiac output (18%) while minimally affecting heart rate and blood pressure.	(45)
**35 healthy individuals**	60 g BC/day Total: 8 weeks	Double-blind, placebo-controlled, parallel study design. Placebo: Whey protein (60g/day in powder form)	The BC group has experienced a significant increase in arm circumference (2.3%) and cross-sectional area (4.2%) compared to whey protein (WP), primarily due to a 5.5% increase in skin and subcutaneous fat (SSF).	(46)
**28 soccer players**	3.2 g BC/day Total: 6 months	Double-blind, placebo-controlled crossover study design. Placebo: Milk (3.2g/day in powder form)	BC supplementation significantly increased immunoglobulin G (IgG) by up to 94% and reduced inflammatory markers (IL-10, TNF-α) after exercise compared to placebo.	(47)
**27 basketball players**	3.2 g BC/day Total: 24 weeks	Randomized, parallel, placebo-controlled study design. Placebo: Milk (3.2g/day in capsule form)	There were no notable impacts on the dynamics of immune system function indicators.	(48)
**9 athletes**	No dose information Total: 10 days	Crossover, double-blind and placebo-controlled study design. Placebo: Skim milk(25g/day in powder form)	When compared to skim milk powder, it did not alter any post-exercise immune variables.	(49)
**20 trained athletes**	3.2 g BC/day Total: 6 months	Randomized, parallel, placebo-controlled study design. Placebo: Milk (3.2g/day in capsule form)	BC supplementation significantly reduced oxidative stress markers (TBARS) compared to placebo (p<0.01).	(50)
**8 healthy individuals**	10 g placebo +37.5 g Zinc 10 g placebo + 10 g BC 37.5 g Zinc + 10 g BC 10 g placebo + 10 g placebo Total: 14 days	Double-blind, placebo-controlled crossover study design. Placebo: Isoenergetic and isomacronutrient milk-protein Concentrate (10g/day + 10g/day in capsule form)	Zinc carnosine (ZnC) and colostrum supplementation effectively mitigated the exercise-induced 3-fold increase in gut permeability by 70%.	(51)
**10 healthy individuals**	20 g BC/day Total: 14 days	Randomized, counterbalanced, placebo-controlled study design. Placebo: Isocaloric and isomacronutrient 63% skim milk, 37% milk protein (20g/day in powder form)	Protected enterocytes in moderate temperatures but not in extreme heat.	(52)
**18 healthy individuals**	20 g BC/day Total: 14 days	Double-blind, placebo-controlled, randomized, crossover study design. Placebo: Isoenergetic and isomacronutrient milk protein concentrate (20g/day in powder form)	Reduced markers of exercise-induced intestinal damage compared to a placebo (191% Placebo to 101% BC). Lactulose/rhamnose ratio were significantly lower in BC group, as representing the intestinal barrier integrity/permeability (148% to 243%)	(2)
**7 trained individuals**	1.7 g BC/day Total: 7 days	Double-blind, randomized, placebo-controlled crossover study design. Placebo: Corn flour (1.7g/day in powder form)	BC supplementation has no effect on highly trained endurance athletes or untrained individuals	(53)
**Adult individuals**	20 g BC/day Total: 14 days	First study: Randomized, double-blind, placebo-controlled study design. Second study: Cohort and observational. Placebo: Maltodextrin (20g/day in powder form)	Significant increase in serum IGF-1 (17%) and saliva IgA (33%)	(1)
**10 highly-trained cyclists**	10 g BC/day Total: 8 weeks	Randomized and controlled study design. Placebo: Whey protein (10g/day in powder form)	Improved cycling performance through the maintenance of testosterone levels and modulation of autonomic activity.	(3)
**Healthy individuals**	5 different trials	Retrospective study design.	Reduction in upper respiratory tract symptoms (URS) in exercising adults (URS days 44%, URS episodes 38%)	(4)
**31 Healthy individuals**	20 g BC/day Total: 58 days	Double-blind, randomized, placebo-controlled study design. Placebo: isoenergetic/isomacronutrient skimmed milk powder and milk protein concentrate (20g/day in powder form)	Enhanced immunity and greater skinfold thickness response in BC group (0.22-0.42 to 0.06-0.16)	(54)
**40 Healthy individuals**	60 g BC/day Total: 8 weeks	Randomized, double-blind, parallel study design.	The colostrum group increased leg press strength from 121 kg to 145 kg (a 19.8% increase), while the whey protein group increased from 143 kg to 151 kg (a 5.6% increase). This represents a 2.8% greater increase for the colostrum group compared to the whey protein group (p = .026).	(55)

Cold symptoms that may occur due to sweating and immune suppression after high-effort workouts can lead to a decrease in performance. BC consumption is critical to protect against colds or viral diseases (56). Because of the Lf it contains, BC not only intervenes in cold symptoms (57) but also supports the immune system by preventing the proliferation of viruses (20–22) (**Table 1**). In the event of a possible disease, Lf support provided by BC consumption increases the response of T-cells by inducing the activity of natural killer cells (58, 59). In this way, it allows the treatment process to be enhanced. Lactoperoxidase (LPO), an antibacterial enzyme, inhibits bacterial metabolism and exhibits toxicity towards various bacterial types, while also possessing antifungal and antiviral activities (60). BC, rich in both LPO and Lf concentrations, can strengthen the immune system of athletes and prevent diseases that may cause a decline in their fitness charts.

BC has a richer nutritional value than regular milk (61). One of these riches is in terms of carbohydrates (62). Oligosaccharides and conjugated *N*-glycans are also included in this family (63). These bioactive components have important potential as they are abundant in BC, show prebiotic properties, and provide selective growth in the intestinal microbiota (24–26) (**Table 1**). Athletes must maintain their intestinal health, especially those engaging in high levels of physical activity. The prebiotic components contained in BC can prevent possible intestinal diseases by supporting athletes’ intestinal health. Additionally, these ingredients’ benefits to the digestive system can increase the efficiency of nutrition programs by allowing athletes to absorb the nutrients they consume more effectively. Therefore, athletes’ regular consumption of BC may be an important step in protecting intestinal health, ensuring more effective absorption of nutrients. This, in turn, can support athletes’ overall health and performance and prevent potential gut problems.

## 3 Potential use of colostrum supplementation in sport and therapeutical or supportive effects

Certain compounds of colostrum are highlighted with their activity in gastrointestinal regions. For instance, certain transforming growth factors that are found in the colostrum have potential roles in gastrointestinal diseases (64). TGF-α can stimulate the secretion of mucin, and enhance regulation of intestinal epithelium by inducing cellular growth and differentiation. TGF-β can also show similar activity to TGF-α. In addition, colostrum can also show anti-inflammatory and antibacterial activity in the region. An *in-vitro* study performed on Caco-2 cells indicated these activities by colostrum (65). The results investigate the antibacterial activity of colostrum and indicate the potential to reduce bacteria-mediated inflammation by decreasing the increase of IL-8 (to back into the control levels in Caco-2 cells, 1 fold increase to 0.5 fold increase in HT29 cells when compared to control levels). Such findings lead to the discussion of the potential application of colostrum in treating or protecting against potential gastrointestinal damage during exercise.

A double-blind crossover study was performed to investigate the activity of BC in intestinal injury protection from exercise (41). During the experiment, 12 healthy males consumed BC for 14 days, and certain trials and blood tests were performed. Also researchers have reported that the exercise-based intestinal injury was determined with the levels of 1-FABP, and the result indicated that BC administration was able to slow down the increase of 1-FABP (placebo group was 194% and the colostrum group was 134%). Another study examined the role of BC administration in gut permeability during heavy exercises. 12 participants were involved in a double-blind crossover study and consumed BC for 14 days. The gut permeability test showed that both groups had a similar level of permeability during the start of the study. In the following days of the exercise, BC managed to decrease the permeability increase by nearly 80% (66).

As discussed, BC can enhance immune regulation and response, anti-pathogenic activity, and protect the gastrointestinal region. As a matter of fact, when these activities are combined, it can potentially lead to a direct positive effect on performance and post-workout recovery (**Table 2**) (66). Another situation that draws attention at this point is the effect of BC consumption on individual performance depending on the consumption of athletes. in a study (34), a controlled BC consumption of hockey players was recorded and then the results were examined, and the results were reported to significantly increase their sprint and jumping performances (2.3%). Additionally, a similar study showed that BC consumption increases jumping performance and also has a significant effect on anaerobic power (35).

As for its effects in long-term competitions, it has been observed that the performance of cyclists who were subjected to a 2-hour ride increased significantly in terms of time after controlled BC consumption. Moreover, it is seen that it can affect the mass development in the athletes’ body by regulating testosterone levels in the body and modulating autonomic activity (3, 36). A study investigated the effect of BC consumption on endurance in running performance, recovery, and plasma insulin-like growth factor 1 levels (35). 51 males performed 8 weeks of running training with two groups; one group consumed 60 g/day BC, and the other group consumed whey powder as a placebo group. The results were significant only for the recovery, as it managed to improve performance (1.5% and 2.3% increase) in the recovery period.

The enhancement on recovery and performance also directly influences muscle gain during the workouts. Since the time of recovery and injury rates are expected to decrease with BC consumption, athletes and sportspersons can enhance their required muscle gain over time by increased performance. Additionally, BC can also directly affect muscle gain, as its components can influence body mass increase positively. For instance, 22 random people were recruited to analyze the effect of BC supplementation on body composition and performance (10). The participants were divided into two groups; 9 people consumed nearly 20g of BC for 8 weeks, and the rest were placebo. Even though there are slight differences between the two groups in terms of exhaustion from exercise, and performance, the only significant differences were observed in the BC-consumed group’s bone-free lean body mass, as the BC-consumed group showed a mean increase of 1.49 kg.

As mentioned in this review article, BC can have various therapeutic effects due to the bioactive elements it contains in a study concluded that BC supplementation increases muscle development in athletes (**Table 2**). From this perspective, it can be used to increase overall muscle mass. One potential compound behind the enhanced muscle gain by BC is IGF-1 since it can help to increase muscle gain by inducing the growth of muscle cells (67). It has been emphasized that muscle size increases are directly proportional to protein synthesis and degradation, of which IGF-1 is known to be capable of altering these processes (68). By that, IGF-1 shows primary mechanisms in modulating muscle size and function and is tightly associated with muscular endurance parameters (69). A recent research investigated the changes in IGF-1 levels in BC supplementation (70). 90 participants were involved as far as the final analysis, in which half of these participants consumed BC. The serum levels showed that the BC-consumed group had increased levels of IGF-1 (*p = 0.02*). Additionally, the incidence rate of diarrhea was also investigated, which was lower in the BC group (27%) when compared to the control group (47%).

In addition, one of the potential positive effects of BC consumption is that it regulates heart function by increasing IGF-1 levels, affecting stroke volume (14%) and cardiac output (18%) with minimal impact on heart rate and blood pressure (45). Another benefit that BC consumption can provide to athletes is “increasing testosterone levels,” which can help athletes achieve more effective results during training and observe positive changes in muscle mass, especially in a short time (3). In a study conducted on female athletes, it was reported that oxidative stress and inflammation in their bodies decreased (p<0.01), and iron homeostasis in their bodies was positively affected as a result of regular consumption of 3.2g BC daily for 6 months (50).

## Conclusion and future perspectives

This review examined the effects of colostrum on athletes. Clinical studies were conducted, and comparative results were reported. Based on these data, it can be said that colostrum consumption has positive effects on athletes. It has been revealed that it has therapeutic effects with its different bioactive components, especially on disorders caused by different sources such as antiviral, antibacterial, gastrointestinal, and injuries. Thanks to the rich composition of BC, its components can enhance many attributes of the consumer, which makes BC consumption preferred for supplementation in exercise and training. When examined, certain effects of BC are possibly linked together, such as enhancement of performance and recovery, which is also another positive aspect of BC consumption for athletes. Currently, colostrum consumption is increasing, yet not all of its therapeutic effects are fully understood. Further research may uncover new data on this subject, potentially revealing additional effects of colostrum beyond current knowledge. This supportive nutrient, which different audiences as well as athletes can use, stands out with its different effects in different sectors (medical, dermatology, food, etc.). Discovering the potential mechanisms of the bioactive components found in colostrum in the future may encourage its more frequent use in various sectors or the treatment of advanced diseases.

## Author contributions

YY: Conceptualization, Visualization, Writing – review & editing, Writing – original draft. BB: Conceptualization, Writing – review & editing. HD: Conceptualization, Visualization, Writing – review & editing. FE: Conceptualization, Visualization, Writing – review & editing. AB: Writing – review & editing. ME: Writing – review & editing, Conceptualization. SK: Conceptualization, Supervision, Visualization, Writing – review & editing.
